# Global Recommendations for Facial Rejuvenation Using a Hyaluronic Acid and Calcium Hydroxyapatite Hybrid Injectable

**DOI:** 10.1111/jocd.70608

**Published:** 2026-01-21

**Authors:** Maurizio Cavallini, Andre Braz, Daniela Greiner‐Krüger, Sylwia Lipko‐Godlewska, Tapan Patel, Marva Safa, Sophie Shotter, Fernando Urdiales Gálvez, Graeme Kerson

**Affiliations:** ^1^ Department of Operative Unit, Dermatology and Dermatosurgery Centro Diagnostic Italiano Hospital Milan Italy; ^2^ Dermatología Láser Cosmiatria Clinic Rio de Janeiro Brazil; ^3^ Medicorium, Center for Dermatology and Aesthetics Oberursel and Friedrichsdorf Germany; ^4^ Private Practice Cracow Poland; ^5^ PHI Clinic London UK; ^6^ Jouvence Neuchâtel Switzerland; ^7^ Illuminate Skin Clinics Kings Hill UK; ^8^ Aesthetic Medicine, Instituto Médico Miramar Málaga Spain; ^9^ Allergan Aesthetics, an AbbVie Company Marlow UK

**Keywords:** aesthetics, combined, dermal fillers, esthetics, evidence‐based medicine, skin aging

## Abstract

**Background:**

The hybrid injectable HA‐CaHA contains a formulated matrix of crosslinked hyaluronic acid (HA) gel with embedded calcium hydroxyapatite (CaHA) microspheres to increase skin elasticity and hydration as well as promote neocollagenesis, respectively. Clinical practice recommendations for use of HA‐CaHA are limited.

**Aims:**

The objective of this article was to provide global recommendations on safe and effective practices for injecting HA‐CaHA.

**Patient/Methods:**

An expert panel consisting of eight international consultants was invited to discuss and provide best‐practice recommendations on HA‐CaHA patient selection, skin laxity assessment, injection techniques, touch‐up/retreatment practices, combination treatment protocols, and safety considerations.

**Results:**

The expert panel recommended using HA‐CaHA for structural support and soft tissue repositioning in patients with skin laxity, sagginess, and mild to moderate loss of volume and contour. They also recommended defining injection entry points by referring to the line of ligaments and the zygomatic arch and keeping injections in the lateral and thus less mobile areas of the face. Experts advised using a cannula instead of a needle, retreating only if mild to moderate skin laxity recurs, and injecting in small volumes. Measures to minimize potential side effects, including injection site responses, were discussed.

**Conclusions:**

Expert clinical recommendations affirm the safe and effective use of HA‐CaHA to improve soft tissue quality over time.

**Abbreviations**
AE: adverse event
CaHA: calcium hydroxyapatite
fLL: functional ligament line
GAIS: Global Aesthetic Improvement Scale
HA: hyaluronic acid
HA‐CaHA: HArmonyCa
MFVDS: Allergan Midface Volume Deficit Scale
TEAE: treatment‐emergent adverse event

## 1 Introduction

More than 13 million injectable aesthetic procedures were performed globally in 2022, a 39% increase from 2018 [1]. About 4.3 million of these procedures were injections of the facial filler hyaluronic acid (HA), and 350 000 were injections of calcium hydroxyapatite (CaHA) filler [1]. Injectables containing HA and CaHA have been widely used to reduce facial wrinkles, add volume, and rejuvenate the skin. Previous studies have shown that HA increases skin elasticity and hydration, and CaHA improves soft tissue quality by promoting new collagen production [2, 3, 4]. Both HA and CaHA result in neocollagenesis in the injected areas, but CaHA does so more robustly, actively remodeling the dermal extracellular matrix [2, 5, 6].

HA‐CaHA (HArmonyCa; Allergan Aesthetics, an AbbVie Company, Irvine, CA, USA) is a novel hybrid injectable containing a formulated matrix of crosslinked HA gel with embedded CaHA microspheres that has been approved for use in more than 30 countries [7, 8, 9]. HA‐CaHA consists of 20 mg/mL crosslinked sodium hyaluronate gel with embedded CaHA microspheres (25–45 μm), plus lidocaine hydrochloride (0.3% w/v) to reduce pain during injections, supplied in a prefilled sterile syringe [7, 8, 9].

Based on the results of preclinical and clinical studies [10, 11, 12, 13, 14, 15, 16, 17], HA‐CaHA can improve facial signs, such as laxity, sagginess, as well as mild to moderate loss of volume and contour, by stimulating new collagen production. The matrix of HA and CaHA provides a dual effect: structural support for facial soft tissue augmentation [18, 19, 20, 21], and fibroblast proliferation for neocollagenesis [19, 20, 21].

Manufacturer‐provided training on injecting HA‐CaHA is offered worldwide. However, due to the relative novelty of HA‐CaHA compared with other injectable products, published literature, including clinical practice recommendations, is limited. The objective of this article is to provide global recommendations on safe and effective practices for injecting HA‐CaHA. Guidance for patient selection, skin laxity assessment, injection techniques, touch‐up/retreatment practices, combination treatment protocols, and safety considerations is discussed. Case study examples are provided to illustrate the recommendations of the experts.

## 2 Materials and Methods

An expert panel/author team, comprising eight international physician advisors, was invited by the sponsor to discuss guidelines and recommendations on the use of HA‐CaHA. These physician advisors were selected based on their expertise in performing aesthetic procedures, experience with the use of HA‐CaHA in clinical practice, and global practice locations. The expert panel qualitatively assessed the following categories of HA‐CaHA treatment initially identified by a literature search using manufacturer training materials, the latest clinical data, published expert opinion, and study publications: HA‐CaHA patient profile, skin laxity assessment, injection techniques, touch‐up/retreatment practices, combination treatment, and safety considerations. Guidelines and recommendations on the use of HA‐CaHA provided by the expert panel were summarized.

## 3 Results

### 3.1 Effectiveness and Safety Data Overview

Key clinical trials highlight the effectiveness of HA‐CaHA along with its favorable safety profile [10, 11, 14, 15, 16]. A retrospective Brazilian study (*N* = 403) that assessed the long‐term (≥ 12‐month) safety of HA‐CaHA in adults who received at least 1 treatment showed that HA‐CaHA was well tolerated [15]. Close to half (46.4%) of the participants enrolled in this study had ≥ 18 months of HA‐CaHA exposure [15]. Overall, there were low rates of treatment‐emergent adverse events (TEAEs) related to treatment, which included edema, product accumulation because of injection technique, and skin induration. TEAEs were mostly mild in severity, and no unexpected TEAEs were reported [15]. HA‐CaHA was also well tolerated in a retrospective study that assessed long‐term safety in adults (*N* = 104) treated in multiple facial areas at least 18 months before evaluation [16]. There were no signs of skin irregularities (e.g., nodules/lumps, erythema, hematoma, necrosis) on visual examination or dermatoscopic photography. High‐frequency ultrasound found no evidence of treatment‐related inflammation, granulomas, or other TEAEs [16].

The favorable safety profile of HA‐CaHA was affirmed in two prospective studies. These studies also demonstrated the effectiveness of HA‐CaHA for improving facial soft tissue. A single‐center quasi‐experimental Brazilian study was conducted in 15 patients who received subcutaneous injections of HA‐CaHA in the subzygomatic region [13]. The study had a 120‐day follow‐up period [13]. At 120 days after treatment, 20% of these patients had an exceptional improvement, 20% were “very improved,” and 60% were “improved” according to the Subject Global Aesthetic Improvement Scale. Only one TEAE occurred (papule, resolved by gentle extraction), and there were no vascular occlusive events, infections, or signs of granuloma formation on clinical exam or ultrasound [13]. In a nonrandomized, single‐session study in 15 patients, HA‐CaHA was injected with a 23‐G cannula (retrograde threads, 1.25 mL per side) in the preauricular region [14]. This study found significant (*p* < 0.0001) increases in volume and facial‐tension vectors on both sides of the preauricular region at day 180 after treatment. Firmness increased at day 60, which was confirmed on day 90 and reached peak effect between days 90 and 180, based on elastography images. These results suggest the formation of new collagen fibers. No unexpected or serious TEAEs occurred. Most patients experienced mild redness and inflammation, which typically resolved within 48 h without intervention [14]. These are typical short‐term reactions for an injectable [4].

Two recent prospective studies have investigated the effectiveness and safety of HA‐CaHA for midface soft tissue augmentation in adults with moderate to severe midface volume deficits [10, 11]. In a multicenter, evaluator‐blinded, randomized, parallel‐group study (*N* = 170), a significantly greater percentage of participants treated with HA‐CaHA reported ≥ 1‐grade improvements from baseline in the Allergan Midface Volume Deficit Scale (MFVDS) at month 3 compared with no‐treatment controls (86.8% vs. 18.5%, respectively; *p* < 0.0001) [10]. A total of 18.0% of participants treated with HA‐CaHA reported treatment‐related TEAEs [10]. Most were injection site related (e.g., bruising and pain) [10]. In an open‐label, postmarketing study (*N* = 140), the majority of participants treated with HA‐CaHA reported a ≥ 1‐grade improvement from baseline in MFVDS, with results lasting through 12 months (82.8% and 65.9% at 1 and 12 months, respectively) [11]. Most participants reported improvements as measured by the FACE‐Q and Global Aesthetic Improvement Scale (GAIS) [11]. Similar to the other prospective midface study, 37.1% of participants reported treatment‐related TEAEs, and the most common treatment‐related TEAEs were injection site pain and mass [11].

Based on the current medical literature described above, as well as their own clinical experience, the authors provide recommendations for patient selection, skin laxity assessment, injection techniques, touch‐up and retreatment, combination treatment protocols, and safety. Two case study examples are also provided to highlight the authors' recommendations.

### 3.2 Patient Selection Recommendations

Previous literature has reported guidelines for selecting ideal patient candidates for HA‐CaHA, which are summarized in Table 1 [9, 12, 22, 23, 24]. These guidelines report that typical candidates consist of adults with laxity, facial sagginess, and mild to moderate volume or contour loss [9]. As a guide for assessing patients, the authors suggest using the functional ligament line (fLL) of the face, an anatomic landmark initially described by Cotofana and Lachman [25]. The fLL refers to an imaginary line connecting the 4 major ligaments of the face that divides the face into medial and lateral sections, delimiting the movable part of the face from the fixed part of the face [26, 27]. The authors suggest that patients who need structure and soft tissue repositioning in the middle and lower face may benefit from HA‐CaHA injected posteriorly to the fLL [26]. In contrast, patients with severe or pronounced laxity, contour loss, volume deficiencies, or other signs and symptoms may merit a different first line of treatment, such as surgery or other modalities (fillers, laser devices, microfocused ultrasonography, radiofrequency, or combined treatment strategies) [9, 22, 23, 24]. HA‐CaHA is contraindicated in active inflammation and infection, skin diseases, and abnormal skin conditions (Table 1).

**TABLE 1:** Patient profile recommendations.

**Recommended selection criteria**
Adults with facial signs that include skin laxity, sagginess, and mild to moderate loss of facial volume or contour [9]
○ Signs that best indicate treatment suitability: lack of skin firmness, presence of “accordion” or radial cheek lines especially during smiling, lateral cheek sagginess or laxity resulting from atrophy, jowls, presence of nasolabial folds, acne scarring and coarse‐pore skin, sun‐damaged skin, midface volume loss, and atrophic skin ○ Potential advantages of hyaluronic acid–calcium hydroxyapatite (HA‐CaHA): hybrid filler appropriate for a broad range of facial signs; ability to be combined with other modalities to treat the full face
No severe facial aging that is better addressed by surgery or additional treatment modalities [9, 12, 22, 23, 24]
No known sensitivity or contraindication to lidocaine or amide‐type local anesthetics, steroids, or any product components; no history of anaphylactic reaction, multiple severe allergies, or both [7]
No skin diseases (especially inflammatory or infectious skin diseases), abnormal skin conditions, or infection or inflammation at or near the treatment site [7]
No susceptibility to keloid formation, hypertrophic scarring, or inflammatory skin conditions [7]
No active herpes infection, autoimmune disease, impaired wound healing due to systemic disorders, medicinal drugs, or unhealthy or poorly vascularized tissue; no prolonged bleeding or tissue healing due to medical conditions or medicinal drugs [7]
Not pregnant or breastfeeding [7] No recent or impending dental procedures (postpone HA‐CaHA until at least 15 days have passed) [9]

Abbreviation: HA‐CaHA, hyaluronic acid–calcium hydroxyapatite.

### 3.3 Skin Laxity Assessment Recommendations

The authors recommend evaluating signs of laxity and sagginess before and after HA‐CaHA to assess both treatment need and effectiveness in repositioning soft tissue and tightening the skin. Table 2 summarizes different methods to assess skin laxity based on the published literature [28, 29, 30, 31, 32]. The “pinch test” (i.e., pinching the skin to gauge its distensibility or thickness and its detachment from deeper planes of tissue) has been reported to be a useful method for assessing the laxity of the area to be treated as well as the effectiveness of the treatment [12, 28, 29, 33, 34]. The authors propose tilting the patient's face and pinching the buccal or lateral area of the face to help establish the injection plan for repositioning or volumizing [28, 29]. Referring to the fLL as an anatomic landmark is also recommended to help establish the injection plan [26, 27]. Other recommendations for assessing skin laxity include digital photography and ultrasonography (Table 2).

**TABLE 2:** Recommended methods for assessing skin laxity.

**Physical/visual examination**
Tilt patient's face and use pinch test to detect laxity and sagginess. It is important to define the functional line of ligaments [35]. The ideal area for the administration of hyaluronic acid–calcium hydroxyapatite (HA‐CaHA) is the fixed part of the lateral cheek. Use the line of ligaments as an anatomic landmark to determine the sequence of injections needed to achieve repositioning or volumizing (first injecting the lateral face and thereby repositioning the medial face)
Pinch‐test the cheeks or lateral area of the middle/lower face to identify laxity based on extent of skin distensibility or thickness and detachment from deeper planes of tissue [12, 28, 29]
Observe other indications of laxity: marked nasolabial or mentolabial folds, presence of prejowls (jowling), accordion lines, and loss of contour in lower face [30, 31, 32]. All these indications of laxity are a consequence of the atrophy in the lateral cheek
**Photography**
Consider evaluating the effectiveness of HA‐CaHA by assessing photos and videos of the patient before and after treatment, fully smiling with molars visible, both in a neutral position and tilted downward at a 30° angle, captured from front, oblique, and lateral views
**Sonography**
Use ultrasound to detect and assess the characteristics of previously injected filler materials, to evaluate for signs of elastosis, and to observe signs suggestive of new collagen [14, 36]

Abbreviation: HA‐CaHA, hyaluronic acid–calcium hydroxyapatite.

### 3.4 Injection Technique Recommendations

Based on the published literature, the authors provide several recommendations on HA‐CaHA injection techniques, depth, and associated volumes, which are summarized in Table 3 [9, 12, 37]. The authors recommend to use a cannula instead of a needle and to avoid injections into highly mobile areas (e.g., lips, periocular region) [7]. Boluses and large volumes are not recommended. Injection into the superficial subcutaneous layer is preferred, using fanning or retrograde linear threading. Gentle massage and molding of the product in the treated area ensure even distribution and conformity with tissue contour.

**TABLE 3:** Recommendations for injection technique and volume for hyaluronic acid–calcium hydroxyapatite (HA‐CaHA) [7, 9, 12, 37].

**Technique**
Fanning or retrograde linear threading is the preferred injection method, using a cannula
Linear retrograde using a small amount of product
Preferred areas of injection include the lateral cheek, preauricular area, submalar area, and mandibular ramus (angle and body) and line
Stop product flow approximately 1 cm before withdrawing the cannula to prevent leakage into subepidermal skin layers
Gently massage and mold the product in the treated area to ensure even distribution of filler and to mold the gel to the tissue contour
Injections into lips, perioral, periocular, and glabellar regions are contraindicated
**Depth**
Inject into the superficial subcutaneous layer
**Volume**
Inject in small volumes; boluses and large volumes are not recommended ○ Amount of product injected per session depends on the clinical condition of the patient and generally ranges between 2.5 and 5 mL ○ Using 0.05–0.1 mL for each line of a fan is recommended for the fanning technique

Abbreviation: HA‐CaHA, hyaluronic acid–calcium hydroxyapatite.

**FIGURE 1:**
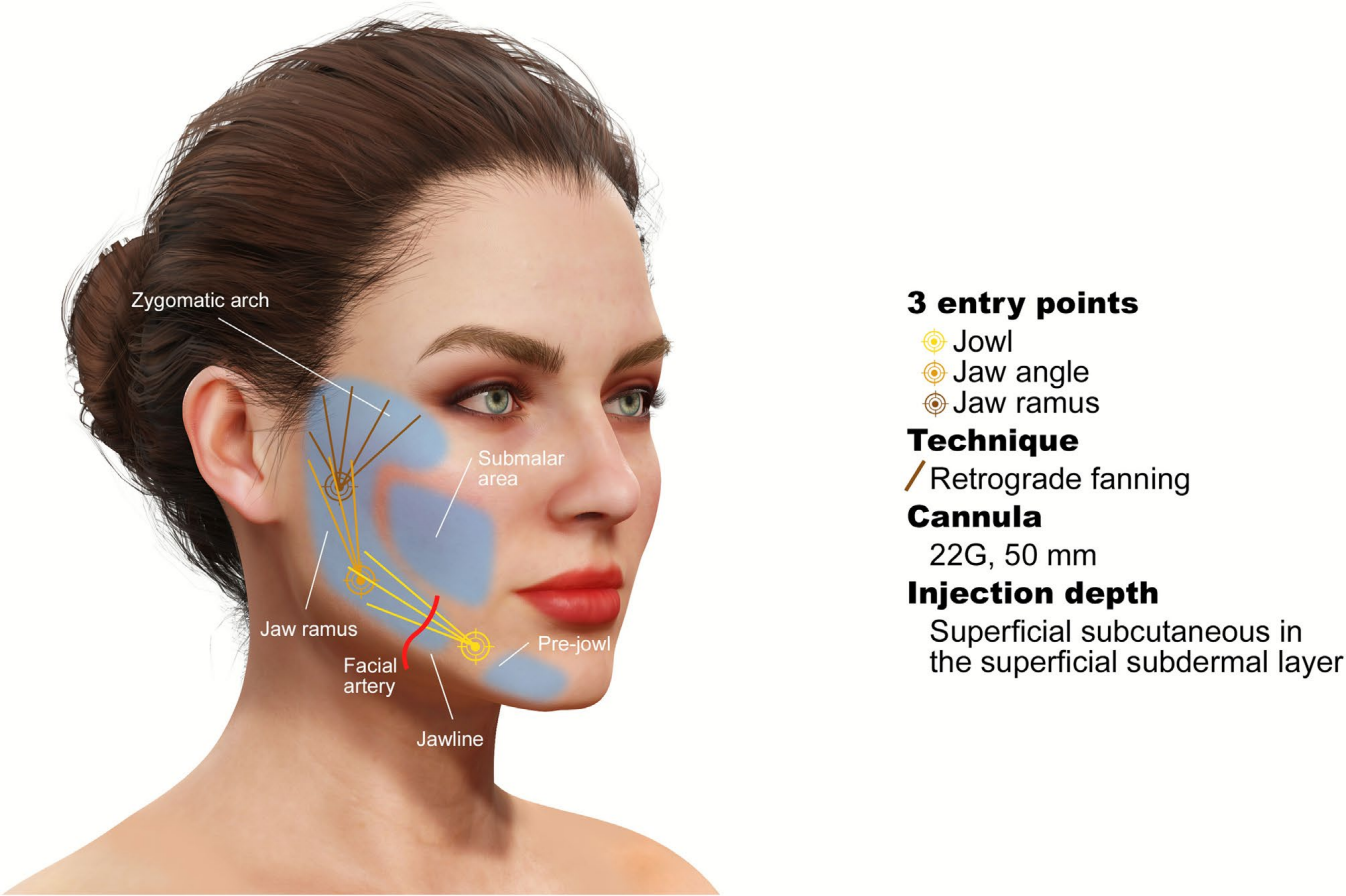
Hyaluronic acid–calcium hydroxyapatite (HA‐CaHA) hybrid injection areas and recommended approach.

The authors propose defining the first, second, and third injection entry points by marking the line of ligaments and the zygomatic arch (Figure 1). Additional anatomical illustrations of recommended HA‐CaHA injection site protocols have been previously published [38]. The authors indicate that the following technique is considered helpful for maintaining the proper injection depth: to pinch the skin, pierce it with a needle at a 45‐degree angle, insert a cannula into the hole at a 45‐degree angle, and then turn the cannula into a 30‐degree angle and inject. Injections are 2.5–5 mL, administered using a linear threading technique, and may be further reduced when using a fanning technique (Table 3). Additionally, the authors recommend initially using smaller doses to gain experience with the performance of HA‐CaHA.

Author experience has shown that keeping injections in the lateral and thus less mobile areas of the face (as shown in Figure 1) results in favorable outcomes and high patient satisfaction. This is probably because lateral skin tightening/repositioning improves medial aspects (e.g., nasolabial folds) that often bother patients. It is recommended to address medial zones with HA monotherapy if additional treatment is needed.

### 3.5 Touch‐up and Retreatment Practice Recommendations

Author recommendations on touch‐up and retreatment practices (Table 4) include assessing patients at about 1 month after treatment to determine the need for touch‐up. In the clinical experience of the authors, most patients may need only one treatment to observe results.

**TABLE 4:** Recommendations for touch‐up and retreatment.

Most patients need one treatment to see results [13]
Collagen stimulation is estimated to occur at weeks 6–8
Use pinch test to identify signs of laxity and sagginess ~1 month after treatment to determine need for touch‐up
Touch‐up can be performed after 1 month if needed
Typically, a retreatment session, if needed, should be conducted after 6 months [15]; expect a lower injection volume for retreatment at this timepoint. Most patients may not require retreatment until after 12 months have passed since the initial treatment

The authors have observed that the effects of HA‐CaHA are seen at 1 month after treatment and increase over time. Based on the literature, practitioners may expect to see maximum results by 3 to 6 months [10, 11, 14]. Therefore, follow‐up visits may be scheduled at 3, 6, 9, and 12 months after treatment to reevaluate skin laxity and volume. The authors advise that treating the patient with an HA filler alone is sufficient when only mild volume loss is observed. Most patients will not need a touch‐up with HA‐CaHA until 12 months have passed since the initial treatment. Retreatment decisions before then should include consideration of the dose of HA‐CaHA that the patient has previously received (i.e., to minimize potential complications, retreatment injections should not result in a total dose greater than 2.5 mL of HA‐CaHA per side). Retreatment is recommended only if volume loss with mild to moderate skin laxity has returned.

### 3.6 Combination Treatment Protocol Recommendations

Based on clinical experience and supported by published literature [39, 40, 41], Table 5 lists recommendations for treating patients with HA‐CaHA along with other products or modalities. Favorable results have been observed by the authors when HA‐CaHA is injected in combination with other products, such as neuromodulators, HA fillers, lasers, and chemical peels, following a panfacial treatment approach [39, 40, 41]. A common practice, in the authors' experience, is using HA‐CaHA with a neuromodulator in the first treatment session, and then following up with energy‐based treatments 1 to 3 months later or with skin quality–enhancing injectables 1 month later, respectively. The authors also suggest performing energy‐based treatments prior to injectables to minimize interactions between superficially placed fillers and deep ablative lasers. Previous reports have shown that this approach can minimize blood contaminations and potentially stimulate fibroblasts [22, 42]. However, this may not always be the case depending on the patient's schedule and availability. Although these insights are based on the clinical experiences of the authors, the timeline ultimately depends on the type of device and the patient's treatment goals. Further clinical studies are needed to explore the optimum sequence and timings of energy‐based devices with HA‐CaHA.

**TABLE 5:** Recommendations for combination treatment.

Hyaluronic acid–calcium hydroxyapatite (HA‐CaHA) can be used in combination with other products, such as neuromodulators, hyaluronic acid (HA) fillers, lasers, and chemical peels, in a panfacial treatment approach with potential superior outcomes to treatment of individual areas [39, 40, 41] ○ Allow ≥ 4 weeks/1 month after HA‐CaHA treatment to perform energy‐based treatments (e.g., ultrasound‐based treatments, deep resurfacing laser, or peeling instruments) [7] ○ In certain cases, waiting ≥ 3 months after HA‐CaHA treatment may be beneficial to allow neocollagenesis [20, 21] ○ Ultimately, the timeline will depend on the type of device and patient treatment goals
Combining or following HA‐CaHA with other treatments is a common practice, especially with VYC filler products
Other treatments are typically administered 1 month after initial HA‐CaHA
Other products may be injected during the session as HA‐CaHA, but not into the same layers

Abbreviations: HA, hyaluronic acid; HA‐CaHA, hyaluronic acid–calcium hydroxyapatite; VYC, Juvéderm Vycross family; Allergan Aesthetics, an AbbVie Company.

### 3.7 Safety Consideration Recommendations

Previous studies report that the most common side effects with HA‐CaHA treatment are mild, temporary injection‐site responses [10, 11, 13, 15], similar to those observed with HA or CaHA, such as redness, swelling, pain, tenderness, and itching [43, 44, 45]. Injection‐site responses occurring with HA‐CaHA typically resolve in 24–48 h, and swelling typically resolves within 1 week [7]. Less commonly reported adverse events (AEs) may include hematoma, seroma, induration, skin pigmentation, persistent nodules, inflammatory reaction, infection, allergic reaction, granuloma, migration, and necrosis [43, 44, 45, 46].

In general, previous guidelines highlight careful patient selection and postprocedure care, as well as proper product preparation and injection techniques, for minimizing or preventing AEs [38, 47]. These guidelines report that strict aseptic practices, injecting HA‐CaHA in small volumes, ensuring precise injection location, and massaging the injected area may minimize complications [4, 38]. The authors provide measures to resolve specific AEs, such as injection site responses, noninflammatory and inflammatory reactions, and vascular occlusions, which are detailed in Table 6. Recommendations and guidelines for the prevention and management of complications with HA‐CaHA have been thoroughly described in a previous publication [38]. Regarding product preparation, the authors caution against the practice of mixing products for injection [48, 49], such as mixing a CaHA filler with an HA filler. Resultant mixtures would not be equivalent to HA‐CaHA, which is specifically manufactured to result in a composite product of embedded CaHA spheres within an HA gel matrix [50, 51]. CaHA filler is suspended in a carrier gel containing carboxymethyl cellulose, and simply agitating with an HA gel would produce a mixture of CaHA, HA, and a carboxymethyl cellulose carrier with unknown rheologic properties different from the original products [49, 51, 52]. As a result, this mixture cannot be expected to produce the same effects as HA‐CaHA [48, 49, 51], which is provided in a prefilled, ready‐to‐use syringe, has a known safety profile, and is backed by clinical data and manufacturer‐provided training.

**TABLE 6:** Recommendations for managing injection‐site responses and adverse events (AEs).

Hyaluronic acid–calcium hydroxyapatite (HA‐CaHA)‐related reactions/AEs	Best practice recommendations
Injection site responses (e.g., redness, swelling, pain, tenderness, itching)	Icing the areas affected by injection‐site responses is recommended For redness, application of ice for a few hours after injection is sufficient If signs of local ischemia (e.g., pain, blanching) appear, stop injection immediately and act appropriately, including massaging the area (in the case of blanching), applying warm compresses, injecting hyaluronidase, considering aspirin, and considering low–molecular weight heparins
Noninflammatory reactions (e.g., nodules)	Nodules likely due to product misplacement or accumulation may be reversible with hyaluronidase treatment, due to its hyaluronic acid (HA) component [38] Flushing with saline is recommended in areas where product misplacement, a nodule, or site mass development is apparent [38] Check all patients for nodules 1 week after treatment; use massage or saline to eliminate [38]
Inflammatory reactions	Anti‐inflammatory antibiotics can be used as treatment [38]
Vascular occlusions	Treatment strategies are similar to the protocol for HA fillers, including applying warm compresses and injecting hyaluronidase [38]. Based on author experience, higher doses of hyaluronidase may be needed for managing vascular occlusions resulting from HA‐CaHA versus HA fillers.

Abbreviations: AEs, adverse events; HA, hyaluronic acid; HA‐CaHA, hyaluronic acid–calcium hydroxyapatite.

## 4 Case Studies

To complement the authors' best practice recommendations, two representative examples of patients who have provided consent for their photographs to be published are described below. In both case studies, the patients presented with moderate to severe skin laxity and were treated with HA‐CaHA according to the approach illustrated in Figure 1. HA‐CaHA also was combined with other products, including onabotulinumtoxinA and HA fillers. The pinch test and photography/imaging modalities, as described in Table 2, were used before and after treatment to evaluate skin laxity and overall changes in facial skin. Posttreatment, the patients showed improvements in skin laxity during their 3‐ and 6‐month follow‐up appointments, which is consistent with previous clinical studies [13, 14].

### 4.1 Case Study 1

**FIGURE 2:**
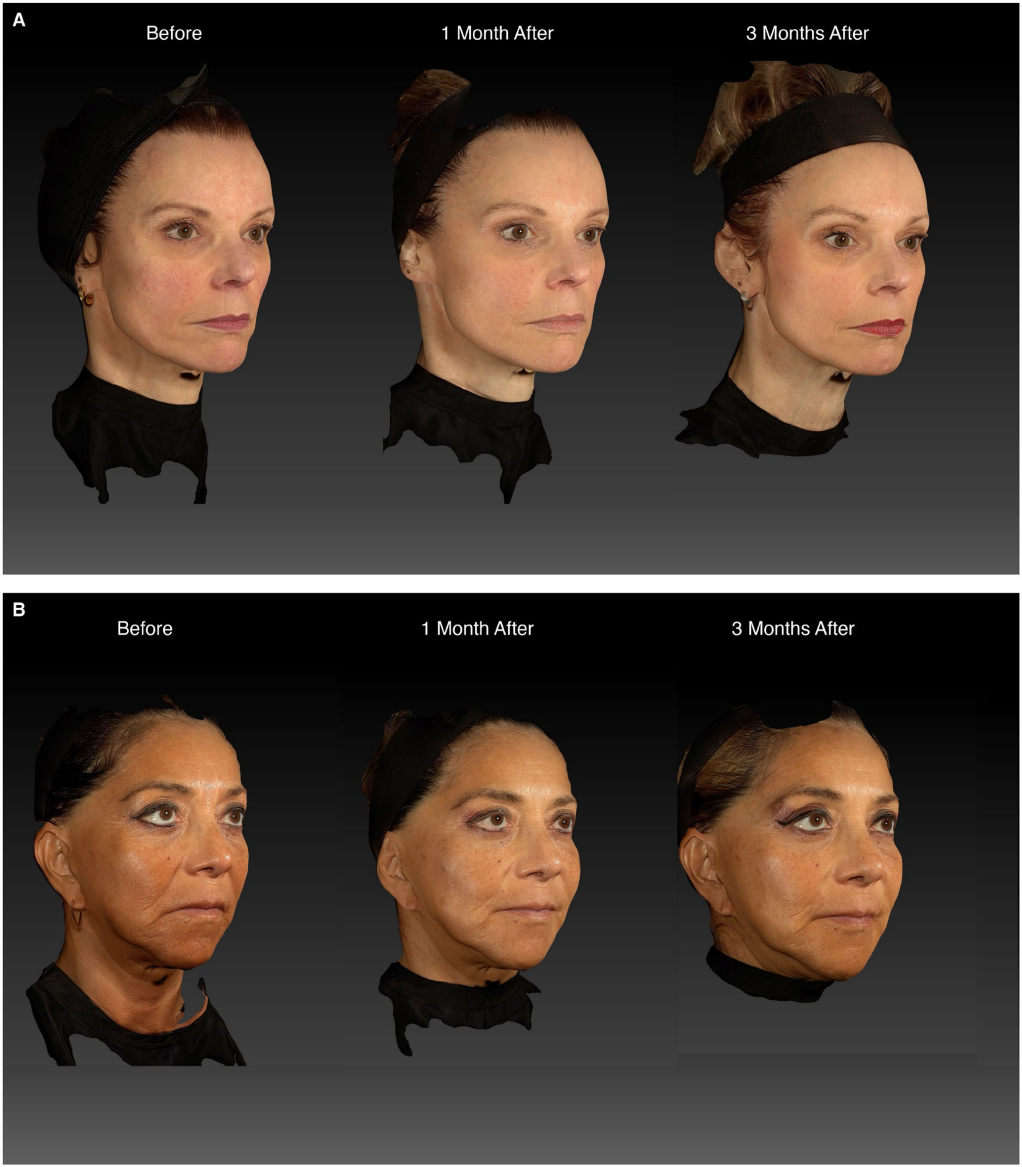
Representative case study photographs illustrating facial rejuvenation using hyaluronic acid–calcium hydroxyapatite (HA‐CaHA) hybrid injectable. (A) A 54‐year‐old woman who received HA‐CaHA, onabotulinumtoxinA, and a hyaluronic acid (HA) filler. (B) A 58‐year‐old woman who received HA‐CaHA and HA fillers. Photographs were taken before, 1 month after, and 3 months after initial treatment. Patient images were provided by Dr. Marva Safa.

A 54‐year‐old White woman from Switzerland was concerned with the appearance of “loose skin” around her jawline and lateral chin (Figure 2A). She presented with severe skin laxity when assessed using the pinch test and moderate facial volume loss. A combination treatment plan composed of HA‐CaHA, onabotulinumtoxinA, and HA filler was formulated. The patient received two syringes of HA‐CaHA (one prefilled syringe of 1.25 mL for each side of the face). HA‐CaHA was injected with a 22‐G, 50‐mm cannula in the zygomatic arch (0.3 mL), preauricular area (0.3 mL), jaw angle along the mandibular bone (0.4 mL), and lateral chin (0.2 mL of HA‐CaHA was delivered from the angle of the mandible using a fanning technique). The patient also received 64 units of onabotulinumtoxinA in the upper face and 2.0 mL of the HA filler VYC‐17.5 L (Juvéderm Volift with lidocaine; Allergan Aesthetics, an AbbVie Company, Irvine, CA, USA) to correct nasolabial folds. The patient received an additional 1.0 mL of VYC‐17.5 L 1 month after initial treatment as touch‐up treatment for the nasolabial folds. At her 3‐ and 6‐month follow‐up appointments after initial treatment, the patient showed improvements in facial skin laxity (i.e., more rapid recoil using the pinch test), skin quality and texture (better light reflection), and facial contour. Furthermore, the patient showed less facial volume loss based on photonumeric assessments with Vectra imaging taken at these timepoints. The patient reported high satisfaction with treatment outcomes. There were no AEs reported immediately posttreatment, and at 1, 3, 6, and 9 months posttreatment.

### 4.2 Case Study 2

A 58‐year‐old Latin American woman from Peru was concerned with the overall appearance of her facial skin, specifically the presence of jowls that made her feel “old” and “sad” (Figure 2B). She presented with moderate skin laxity in the lower face when assessed using the pinch test, jowling, poor skin quality and texture, and slight facial volume loss. A combination treatment plan composed of HA‐CaHA and HA fillers was formulated. The patient received two syringes of HA‐CaHA (one prefilled syringe of 1.25 mL for each side of the face). HA‐CaHA was injected with a 22‐G, 50‐mm cannula in the preauricular area (0.5 mL), angle of the mandible (0.2 mL), and lateral chin (0.5 mL was delivered along the jawline using a fanning technique). HA‐CaHA was not injected in the zygomatic arch because the patient did not present with skin laxity in this area and the patient also had sufficient volume and anterior projection. The patient also received 1.0 mL each of the following HA fillers: VYC‐20 L (Juvéderm Voluma with lidocaine; Allergan Aesthetics, an AbbVie Company) in the midface and VYC‐15 L (Juvéderm Volbella with lidocaine; Allergan Aesthetics, an AbbVie Company) in the upper lip, oral commissure, and marionette lines. The patient received an additional 1.0 mL each of VYC‐20 L and VYC‐17.5 L 1 month after initial treatment, then another 1.0 mL of VYC‐15 L 3 months after initial treatment as touch‐up treatments. At her 3‐ and 6‐month follow‐up appointments after initial treatment, the patient showed improvements in facial skin laxity (i.e., more rapid recoil using the pinch test), jowling, skin quality and texture (better light reflection), and facial contour. Furthermore, the patient showed less volume loss and improvements in fine lines and wrinkles in the lateral chin and marionette lines based on photonumeric assessments with Vectra imaging taken at these timepoints. The patient reported high satisfaction with treatment outcomes, especially improvements of the jawline and prejowls, as well as skin quality and texture. There were no AEs reported immediately posttreatment, and at 1, 3, 6, and 9 months posttreatment.

## 5 Discussion

Clinical studies and guidelines have shown that HA‐CaHA, either used alone or in tandem with other modalities, can address a variety of signs and deficits in facial skin, including skin laxity, sagginess, and mild to moderate loss of facial volume or contour [10, 11, 12, 13, 14, 15, 16, 17]. Furthermore, HA‐CaHA has a favorable safety profile [10, 11, 13, 14, 15, 16]. The most commonly reported side effects with HA‐CaHA treatment are mild, temporary injection‐site responses similar to those observed with HA or CaHA [10, 11, 13, 14, 15, 16]. Because HA‐CaHA is a relatively novel injectable product, more published literature summarizing current data and clinical practice recommendations is needed to help clinicians make informed recommendations to patients in order to achieve optimal treatment outcomes.

This article has several limitations, including the off‐label nature of some recommendations and the lack of standardized methods to generate the literature search. Because the literature search was unstructured, the included publications may limit the generalizability of findings. Limitations of some of the currently available studies cited in this article include small sample sizes, short follow‐ups, and lack of head‐to‐head trial data. Although existing literature supports a positive safety profile of HA‐CaHA, long‐term safety data regarding HA‐CaHA are needed to comprehensively evaluate the potential risk of delayed complications, such as delayed‐onset nodules, with treatment. Future clinical studies should focus on large‐scale, long‐term, head‐to‐head clinical trials with other injectables.

## 6 Conclusions

This paper provides important information to guide clinicians in the use of HA‐CaHA. These recommendations support clinicians in safely and effectively using HA‐CaHA to improve skin architecture and soft tissue quality over time for an enduring effect in individuals with signs of facial aging.

## Author Contributions

No honoraria or payments were made for authorship. All authors meet ICMJE authorship criteria, including review of article design, collection and review of literature, literature analysis and interpretation, manuscript review and revisions, and final approval of manuscript.

## Funding

Allergan Aesthetics, an AbbVie Company, funded this study and participated in the study design, research, analysis, data collection, interpretation of data, reviewing, and approval of the publication. All authors had access to relevant data and participated in the drafting, review, and approval of this publication. No honoraria or payments were made for authorship. Medical writing support was provided by Regina Kelly, MA, of Peloton Advantage LLC (an OPEN Health company) and funded by Allergan Aesthetics, an AbbVie Company.

## Ethics Statement

The authors have nothing to report.

## Consent

All patients have provided written informed consent for their photos to be published.

## Conflicts of Interest

M.C. is a key opinion leader for Allergan Aesthetics, an AbbVie Company, and a member of the international HArmonyCa faculty. A.B. is a key opinion leader for Allergan Aesthetics, an AbbVie Company, and was a member of the international HArmonyCa faculty and involved in the international experience phase before the product was launched. D.G.‐K. is a key opinion leader and AMI faculty member for Allergan Aesthetics, an AbbVie Company, and was a member of the international HArmonyCa faculty and involved in the international experience phase before the product was launched. S.L.‐G. is a key opinion leader for Allergan Aesthetics, an AbbVie Company, and was a member of the international HArmonyCa faculty and involved in the international experience phase before the product was launched. T.P. is a key opinion leader for Allergan Aesthetics, an AbbVie Company. M.S. is a key opinion leader for Allergan Aesthetics, an AbbVie Company. S.S. is a key opinion leader for Allergan Aesthetics, an AbbVie Company, and was a member of the international HArmonyCa faculty and involved in the international experience phase before the hybrid injectable was launched. F.U.G. is a key opinion leader for Allergan Aesthetics, an AbbVie Company. G.K. is a full‐time employee of AbbVie and may own AbbVie stock.

## Data Availability

AbbVie is committed to responsible data sharing regarding the clinical trials we sponsor. This includes access to anonymized, individual and trial‐level data (analysis data sets), as well as other information (e.g., protocols, clinical study reports, synopses, or analysis plans) as long as the trials are not part of an ongoing or planned regulatory submission. This clinical trial data can be requested by any qualified researchers who engage in rigorous, independent scientific research and will be provided following review and approval of a research proposal and Statistical Analysis Plan (SAP) and execution of a Data Use Agreement (DUA). Data requests can be submitted at any time after approval in the US and Europe and after acceptance of this manuscript for publication. The data will be accessible for 12 months, with possible extensions considered. For more information on the process, or to submit a request, visit the following link: https://vivli.org/ourmember/abbvie/ then select “Home.”
